# Using Normative Language When Describing Scientific Findings: Protocol for a Randomized Controlled Trial of Effects on Trust and Credibility

**DOI:** 10.2196/41747

**Published:** 2022-09-09

**Authors:** Jon Agley, Yunyu Xiao, Esi E Thompson, Lilian Golzarri-Arroyo

**Affiliations:** 1 Prevention Insights Department of Applied Health Science, School of Public Health Bloomington Indiana University Bloomington Bloomington, IN United States; 2 Department of Population Health Sciences Weill Cornell Medicine New York, NY United States; 3 The Media School Indiana University Bloomington Bloomington, IN United States; 4 Biostatistics Consulting Center School of Public Health Bloomington Indiana University Bloomington Bloomington, IN United States

**Keywords:** trust, trust in science, scientific communication, meta-science, RCT, randomized controlled trial, infodemic, COVID-19, misinformation, normative language, meta-cognitive, cognitive, scientific information, credible, credibility

## Abstract

**Background:**

Trust in science and scientists has received renewed attention because of the “infodemic” occurring alongside COVID-19. A robust evidence basis shows that such trust is associated with belief in misinformation and willingness to engage in public and personal health behaviors. At the same time, trust and the associated construct of credibility are complex meta-cognitive concepts that often are oversimplified in quantitative research. The discussion of research often includes both normative language (what one ought to do based on a study’s findings) and cognitive language (what a study found), but these types of claims are very different, since normative claims make assumptions about people’s interests. Thus, this paper presents a protocol for a large randomized controlled trial to experimentally test whether some of the variability in trust in science and scientists and perceived message credibility is attributable to the use of normative language when sharing study findings in contrast to the use of cognitive language alone.

**Objective:**

The objective of this trial will be to examine if reading normative and cognitive claims about a scientific study, compared to cognitive claims alone, results in lower trust in science and scientists as well as lower perceived credibility of the scientist who conducted the study, perceived credibility of the research, trust in the scientific information on the post, and trust in scientific information coming from the author of the post.

**Methods:**

We will conduct a randomized controlled trial consisting of 2 parallel groups and a 1:1 allocation ratio. A sample of 1500 adults aged ≥18 years who represent the overall US population distribution by gender, race/ethnicity, and age will randomly be assigned to either an “intervention” arm (normative and cognitive claims) or a control arm (cognitive claims alone). In each arm, participants will view and verify their understanding of an ecologically valid claim or set of claims (ie, from a highly cited, published research study) designed to look like a social media post. Outcomes will be trust in science and scientists, the perceived credibility of the scientist who conducted the study, the perceived credibility of the research, trust in the scientific information on the post, and trust in scientific information coming from the author of the post. Analyses will incorporate 9 covariates.

**Results:**

This study will be conducted without using any external funding mechanisms.

**Conclusions:**

If there is a measurable effect attributable to the inclusion of normative language when writing about scientific findings, it should generate discussion about how such findings are presented and disseminated.

**Trial Registration:**

Open Science Framework n7yfc; https://osf.io/n7yfc

**International Registered Report Identifier (IRRID):**

PRR1-10.2196/41747

## Introduction

### Trust in Science and Scientists

The “infodemic” [1] accompanying the COVID-19 pandemic [2,3] has reinforced the importance of studying how people perceive information about and from scientific studies. When people are presented with a high volume of information of varying accuracy, we propose that there exists a broad social interest in *people making decisions based on the best available evidence*. Our team’s epistemological framework is that the scientific method is an excellent means of producing evidence. However, the suitability of the scientific method for producing knowledge, in general, does not necessarily mean that all purported scientific findings are of equal weight or that all scientists’ findings or statements are trustworthy [4,5]. Indeed, alongside truly remarkable scientific discoveries during the pandemic, Retraction Watch recently documented its 250th COVID-19 paper retraction, and many of the papers on their list are associated with commonly shared misinformation about the pandemic (eg, the supposed role of 5G networks in spreading COVID-19) [6]. Thus, although we will shortly argue that trust in science and scientists is valuable to understand, it is important not to conflate such trust with a more simplified aphorism such as “trust the science” [7], which (in our view, inappropriately) implies a singular “science” that is inherently worthy of trust.

At the same time, even though trust is defined and measured in a variety of different ways, studies conducted across multiple different populations have identified associations between trust in science and intention to get vaccinated or boosted for COVID-19 [8-12], as well as adherence to other measures to mitigate harm from the pandemic (eg, nonpharmaceutical interventions) [13-17]. Thus, even if the exact nature of the relationship is unclear, it is reasonable to speculate that trust in science and scientists is associated with people’s behaviors during a public health emergency such as COVID-19. We also note that trust in science and scientists is associated with belief in misinformation. Our research team has been studying trust in science and scientists as a complex and multifaceted variable using a conceptualization from Nadelson et al [18]. They validated a 21-item measure that includes multiple conceptual domains related to trust, computed using responses to items such as “We cannot trust scientists to consider ideas that contradict their own,” and “When scientists form a hypothesis they are just guessing” [18]. Using their 21-item measure to compute an aggregated value for trust, we found evidence of strong associations between low trust in science and belief in scientifically unsupported statements (eg, “misinformation”) about COVID-19 [19,20] and opioid overdose and naloxone [21].

We used those findings to develop a digital intervention, very deliberately rejecting approaches focused on the manipulation of trust in favor of creating truthful informational infographics to explain common misperceptions about science (ie, why it is appropriate for scientists to change their minds in response to new evidence) [22]. In a large preregistered, randomized controlled trial, we found evidence that 60 seconds of exposure to one such infographic, which featured an example of changing recommendations around butter and margarine, slightly and significantly increased trust in science and scientists and slightly reduced the likelihood that participants whose trust was thereby increased would endorse COVID-19 misinformation (ie, a mediational, but not direct, intervention effect) [20]. However, we observed no effects on behavioral intentions whatsoever, even though exploratory analyses showed that the trust variable remained associated with behavioral intentions [20].

This difference between the intervention’s effects on beliefs and behavioral intentions may raise questions about the ways in which scientific claims, as well as misinformation, may contain both cognitive and normative elements (which we unpack subsequently) and whether such elements are associated with trust in science and scientists. Furthermore, recent papers have reemphasized the importance of attending to both universals (eg, “trust in science and scientists”) and particulars (eg, “credibility of a specific scientist or claim”) in this area of study [4,5]. Thus, the remainder of this introduction makes the case for rigorously examining a seemingly small—but potentially important—distinction between cognitive and normative claims and the impact of such a difference on universal and particularized trust and describes our plan to do so.

### Cognitive and Normative Scientific Claims

In this study, we will scrutinize the differences between “cognitive” (ie, epistemic) claims of scientific findings and “normative” claims (ie, recommendations). For many scientific endeavors (such as experiments or evaluative studies), a key outcome of the research is 1 or more cognitive claims. A basic example of a cognitive claim is “the current is really strong out by the pier,” which is an assertion about the strength of the current. Importantly, a cognitive claim does not include a recommendation about what we ought to do if the claim is true. Such “ought” statements are instead normative claims, which pertain to what a person *should do* (eg, if it is the case that the current is really strong out by the pier, “...you should not swim there”) [23].

In a consequentialist account of rationality, cognitive claims can be separated from normative or practical claims [23]. We often use others’ cognitive claims to understand the world around us (eg, “we know by trusting what others tell us”) [24]. This trust is necessary because no person has the ability to generate empirical knowledge about everything (given constraints such as capacity, resources, interest, and time). Thus, people frequently make decisions about which and whose cognitive claims to trust [25].

In contrast, normative claims presume a framework of interests (eg, not wanting to swim in dangerous places), but such framing may not always apply to all persons (eg, someone who is at the pier to test a device built to measure current strength). Even if we trust a cognitive claim, we may not trust an associated recommendation [26]. A helpful real-life anecdote can be found in a headline from July 2021 titled “People are dressing in disguise for COVID-19 vaccines, Missouri doctor says” [27]. For the people in that story, the recommendation to get vaccinated was complicated, not because of any explicit disbelief in scientific cognitive claims about the vaccine, but because it did not account for a strong competing interest in not being “ridiculed” by friends, family, or coworkers. Similarly, when physical conflicts—including some resulting in death—erupted across the United States in 2021 over face masks, media coverage suggests the primary concern was not the science behind face masks, per se, but rather whether people *should* or *should not* be wearing them in a particular public space and time [28-31].

We hypothesize that some meaningful percentage of variability in trust in science and scientists, both generally and at the level of specific claims and persons, is attributable to the linguistic entanglement of cognitive and normative claims (eg, in press releases, popular summaries, and social media). In particular, we expect that some portion of mistrust is unrelated to scientific epistemic claims and may instead be explained by perceptions of discordant interests between laypersons and scientists. If that is the case, then we would expect to see reduced levels of trust and perceived credibility in study participants exposed to both a cognitive and normative claim about a study compared to those exposed only to a cognitive claim (ie, “I believe your finding, but I don’t agree with your recommendation”). If we are correct, it may have important ramifications for the way in which scientific findings are reported at multiple levels (eg, abstract, press release, news coverage, and social media).

### Study Objectives and Hypotheses

Our study will draw conclusions by randomizing a large, nationally representative sample of US adults to view a sample social media post that either (1) shares a cognitive claim from a 2020 study on face masks (control group), or (2) shares the same cognitive claim but also includes a normative claim about what people should do, given the cognitive claim, which is also from that study (intervention group; see Methods). We hypothesize the following (see Table 1):

- Hypothesis 1: Overall trust in science and scientists (21-item scale) [18] will be significantly lower in the intervention arm (cognitive and normative claims) than the control arm (cognitive claim only).
- Hypotheses 2-5: The credibility of the scientist who conducted the study, credibility of the research, trust in the scientific information on the post, and trust in scientific information coming from the author of the post [32] will each be significantly lower in the intervention arm (cognitive and normative claims) than the control arm (cognitive claim only).
- Preregistered analyses (without hypotheses): We will study the interaction between the study arm and political orientation for each of the 5 preregistered hypotheses.

**Table 1:** Design table.

Question	Hypothesis (if applicable)	Sampling plan (eg, power analysis)	Analysis plan	Interpretation given to different outcomes
What is the effect of intervention arm assignment on overall trust in science and scientists (21-item scale)?	Overall trust in science and scientists (21-item scale) will be significantly lower in the intervention arm than the control arm.	With 80% power (2-tailed test), this sample will allow us to detect small effects at α=.05 (Cohen *d*=0.14) and at corrected α=.01 (Cohen *d*=0.18) for differences between groups.	ANCOVA^a^, incorporating all 9 listed covariates	A significant effect will be interpreted as evidence that the inclusion of normative language caused the change (if the change exists), regardless of direction.
What is the effect of intervention arm assignment on the credibility of the research, credibility of the scientist who conducted the study, trust in scientific information from the author of the post, and trust in the scientific information on the post?	For hypotheses 2 through 5, each dependent variable will be significantly lower in the intervention arm than the control arm.	With 80% power (2-tailed test), this sample will allow us to detect very small effects at α=.05 (Cohen *d*=0.14) and at corrected α=.01 (Cohen *d*=0.18) for differences between groups.	ANCOVA, incorporating all 9 listed covariates	A significant effect will be interpreted as evidence that the inclusion of normative language caused the change (if the change exists), regardless of direction.
Are there any significant interactions between intervention arm assignment and political orientation on any of the prespecified dependent variables?	N/A^b^	N/A	Linear regression including the interaction between intervention arm and political orientation; the model will include the remaining 8 covariates	A significant interaction will be interpreted as possible evidence that political orientation may mediate or moderate the effect of including normative language in some way, regardless of direction, but that further research is needed.

^a^ANCOVA: analysis of covariance.

^b^N/A: not applicable.

## Methods

### Ethics Approval and Consent to Participate

This study will comply with relevant ethical regulations as outlined and approved by the Indiana University Institutional Review Board (16141) on August 2, 2022. Informed consent will be obtained from participants using electronic agreement to a study information sheet embedded at the beginning of the experiment. Participants will be paid US $1.50 on the successful completion of the study and submission for compensation (see Sampling Plan).

### Design

#### Trial Design

**Figure 1:**
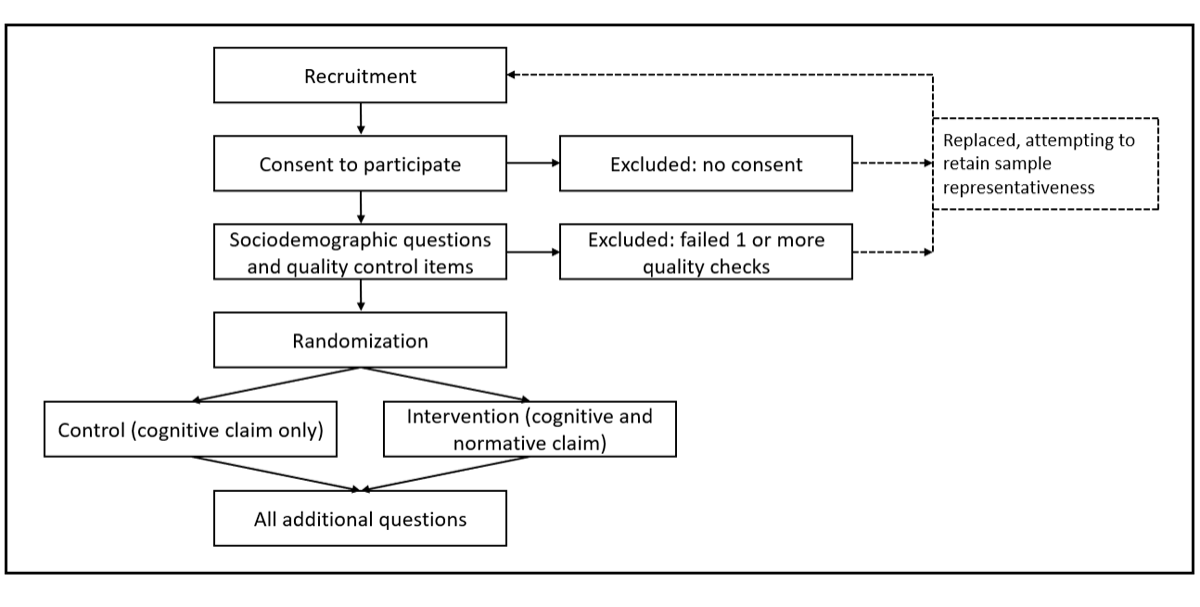
Study design and workflow.

We will conduct a randomized controlled trial consisting of 2 parallel groups and a 1:1 allocation ratio. Our planned study workflow is provided in Figure 1. Allocation will be automated by the Randomizer feature in the QualtricsXM platform [33] and thus, by definition, will be concealed from researchers until after each case has been assigned. However, we note that based on our prior experience, the use of data quality checks and the 2-step survey completion procedures in the Prolific platform can result in unbalanced allocation for a very small portion of the sample as recruitment approaches the planned limit (eg, if the number of usable surveys exceeds the number of surveys solicited, the excess surveys may not be allocated 1:1 but will still be included following sensitivity analysis) [20].

This study will be, in principle, a double-blind study; participants will be unaware that they are being randomized to a condition, and allocation will be done exclusively by software. However, analysts will not be blinded to the meaning of the group assignment variable.

#### Participants and Procedures

This study will solicit participants (n=1500) from the Prolific crowdsourced US representative participant pool, which provides a cross-section of age, sex, and race/ethnicity that mirrors the national US population [34]. This sampling frame also provides the de facto eligibility criteria of being aged ≥18 years and residing in the United States. Studies have found the Prolific platform to produce high-quality research data in general and relative to competing services (eg, Qualtrics and Dynata panels, Amazon MTurk, and CloudResearch) [35,36].

When individuals indicate interest in participating in the study, they will be provided with a link to the experiment, which will be hosted on the QualtricsXM platform. Participants who agree to participate in the study (study information sheet) will complete the first block of questions, which will include sociodemographic items intermixed with screening questions to identify the risk of low-quality data (ie, virtual private network or bot use, inattention, and dishonesty) [37]. Individuals who fail 1 or more checks will be considered ineligible for the study and asked to return the study to the Prolific platform for reselection.

As noted in Figure 1, randomization will occur after all determinations of eligibility to preserve sample composition and allocation. The study will be fully insular (eg, all components occur within the QualtricsXM platform and within 1 “sitting”). To mitigate the potential impact of missing data, participants will not be permitted to advance the survey if they have not answered all questions on a given page. All data will be exported directly from the QualtricsXM platform to a local data file once the study closes, and the code used for data cleaning (see Sampling Plan) will be shared alongside the raw data.

#### Intervention and Control Description

**Figure 2:**
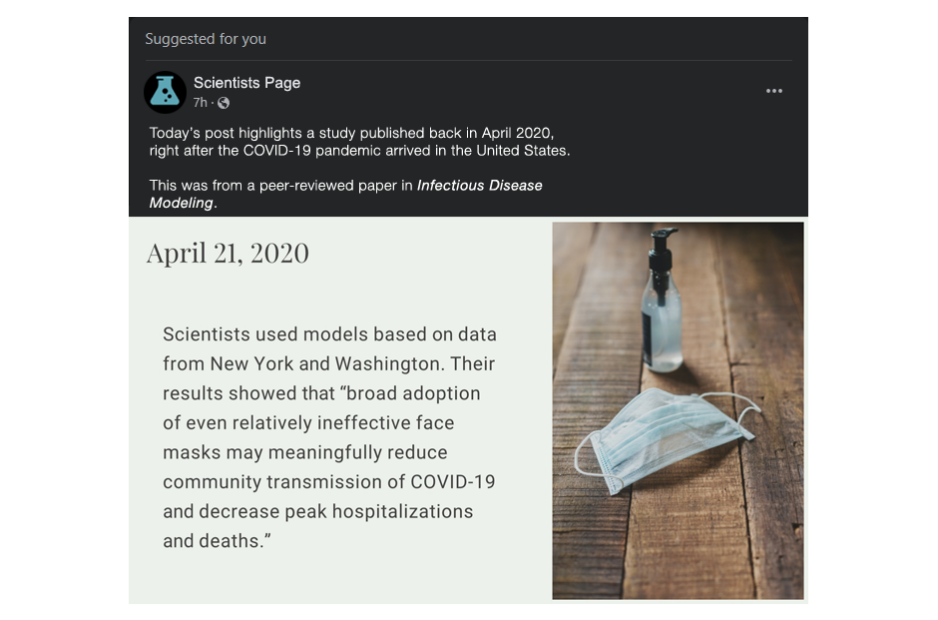
Control image.

**Figure 3:**
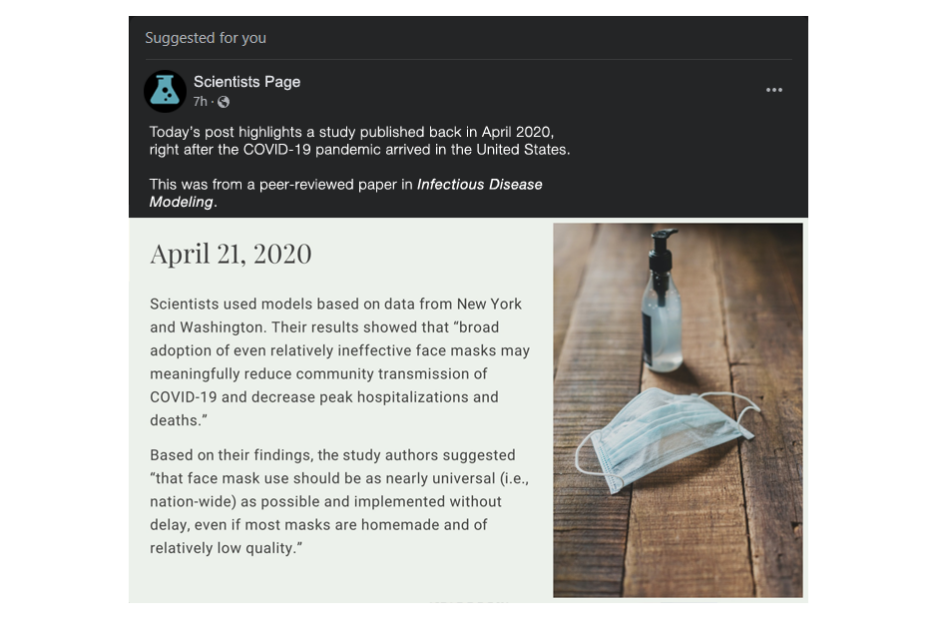
Intervention image.

To isolate the specific effects of normative language in describing scientific findings, we will show participants 1 of 2 fake social media posts depending on the study arm to which they are randomized. The posts will be identical except for the inclusion of normative language in the intervention post. To improve ecological validity [38], both posts will be designed using the formatting parameters for modern Facebook timeline posts as displayed in a web browser using “night” mode (see Figures 2 and 3). The posts will appear as posts “Suggested for you” from a generic science page, which is the primary subheading that Facebook uses when inserting content that is not explicitly followed on a person’s timeline. The lead-in text will read, “Today’s post highlights a study published back in April 2020, right after the COVID-19 pandemic arrived in the United States. This was from a peer-reviewed paper in *Infectious Disease Modeling*.” Both posts will be accompanied by the same generic image of a surgical face mask and a bottle of hand sanitizer.

Additionally, in service of ecological validity, the written content of the graphics will be drawn directly from a highly cited April 2020 paper on face masks for the prospective prevention of COVID-19 [39]. The control post (Figure 2) will feature only a cognitive claim: “Scientists used models based on data from New York and Washington. Their results showed that ‘broad adoption of even relatively ineffective face masks may meaningfully reduce community transmission of COVID-19 and decrease peak hospitalizations and deaths.’”

The intervention post (Figure 3) will include the same cognitive language but also add a normative statement derived from the same source paper. It will add “Based on their findings, the study authors suggested ‘that face mask use should be as nearly universal (i.e., nation-wide) as possible and implemented without delay, even if most masks are homemade and of relatively low quality.’”

Once the image is displayed, participants will not be able to advance the survey for at least 30 seconds (the button to continue the survey will be linked to a timer). A message indicating this delay will be provided on the screen. After participants advance, they will transition to a screen featuring a smaller copy of the image appropriate for their study arm, along with 1 (control) or 2 (intervention) comprehension questions.

- Both arms: (True/False) “In the social media post you read about a scientific study from April 2020, the study authors found that face masks could reduce the spread of COVID-19 as well as lowering hospitalizations and deaths.”
- Intervention arm only: (True/False) “In the social media post you read about a scientific study from April 2020, the study authors recommended that everyone in the US should start wearing masks immediately.”

Any response other than “True” for either question will display a message asking the participant to read the post carefully again, and then participants will be returned to the social media post (no 30-second delay on subsequent viewings), from which they can proceed forward to the comprehension questions again.

### Sampling Plan

#### Sample Size Determination and Power Analysis

As previously described, we will recruit 1500 individuals from the Prolific platform, with equal allocation to study arms. This sample size is currently the largest number of people for which the Prolific platform can assure national representativeness. We made the decision to recruit a large number of participants because we were unable to locate any baseline studies with which to estimate effect size, so we wanted to be conservative. In addition, although the issue of face masks continues to be a point of public contention, the prevalence of public health mandates related to face masks is lower than those in 2020-2021, which we suspect may serve to suppress the effect size relative to what it would be in the midst of more active social discord focused on that topic. With 80% power (2-tailed test), this sample will allow us to detect small effects at α=.05 (Cohen *d*=0.14) and at corrected α=.01 (Cohen *d*=0.18) for differences of trust in science between treatment and control. In keeping with modern inferential statistical recommendations, we will report all *P* values precisely rather than by threshold [40] and provide a balanced interpretation, especially where .01<*P*<.05.

#### Technical Exclusion and Inclusion Procedures

With participant recruitment through a web-based pool, the number of usable responses is specified first (ie, the sample size)—in this case, 1500. When participants complete the survey, they will submit evidence of completion to the researchers, who will then approve payment via the Prolific platform. When an individual is rejected for payment (either at the time of submission for payment or at any other time), they will be resampled and replaced by a new member of the pool who matches their sociodemographic characteristics (eg, age, sex, and race/ethnicity). As a result, the number of participants who accept the survey initially will generally be different than the final sample size. This difference can occur for the following reasons (we provide *rough estimates* of prevalence based on a prior large study with the Prolific platform) [20]:

- Excluded: refusal to participate by declining consent (approximately 0.19%)
- Excluded: failing a quality control check and “returning” the survey (approximately 2.14%)
- Excluded: exiting the Qualtrics platform immediately after consenting to participate (approximately 0.37%)
- Excluded: exiting the Qualtrics platform prior to accessing the intervention, typically, but not always, after being informed of failing a quality control check (approximately 2.88%)
- Included: completing the full study correctly but failing to submit a request for payment (approximately 1.58%)

The overall estimated impact is that is roughly 5.57% of individuals who initially access the survey from the study pool will end up being rejected and resampled in the process of reaching the targeted sample size of 1500. Subsequently, a small number of individuals (roughly 1.58%) may fail to submit for payment but otherwise provide good data; these individuals will be analyzed in the arms to which they were assigned (but as noted earlier, will not be allocated 1:1).

### Analysis Plan

#### Primary Outcomes

The primary outcomes are as follows.

- Hypothesis 1
  Overall trust in science and scientists will be measured by the 21-item scale developed and validated by Nadelson et al [18]. An example item from the scale measures agreement with the statement, “When scientists change their mind about a scientific idea it diminishes my trust in their work.” This scale has demonstrated excellent internal reliability in our previous studies with crowdsourced samples (α>.900) [19-22,41].
- Hypotheses 2 through 5
  Single-item measures of credibility and trust that are specific to the hypothetical social media post and the scientist who conducted the study, from Song et al [32]
  “How credible is the scientist who conducted the study described in the post?” (1=not credible at all to 7=extremely credible); note that this language is slightly different than the original item to avoid ambiguity arising from the potential that a scientist authored the social media post
  “How credible is this research?” (1=not credible at all to 7=extremely credible)
  “I would trust scientific information if I knew it came from this author.” (1=strongly disagree to 7=strongly agree)
  “I trust this scientific information.” (1=strongly disagree to 7=strongly agree)

#### Covariates

The covariates are as follows.

- Familiarity with science will be measured by 1 item asking, “How often do you read science papers or science in the news?” (1=never to 5=always) [32]; this item was suggested in Song et al [32] as being potentially important to consider
- Level of religious commitment (0=low to 10=high), as used in our previous studies [19,20,41]
- Political orientation (0=liberal to 10=conservative), as used in our previous studies [19,20,41]
- Political party (Republican, Democrat, or other), given recent research suggesting divergence between political orientation and party orientation pertaining to face masks [42]
- Race, ethnicity, gender, age (“About how old are you (*in years*)?”), and education level (“What is the highest grade or level of school you have completed, or the highest degree you have received?”) [43]

#### Statistical Analyses

Descriptive statistics of demographics and outcomes will be calculated. We will explore distributions of trust in science (Hypothesis 1) and each of the single-item measures (Hypotheses 2-5).

Data will be analyzed using analysis of covariance (ANCOVA), with the assignment of the study arm (intervention vs control) used as the independent variable and the specified outcomes (depending on the hypothesis) set as the dependent variable. All specified covariates will be included in each model, and assumptions for the analyses will be evaluated (eg, the normality of residuals and linear relationships between covariates and the dependent variable). If assumptions are not met, the research team will explore different transformations or nonparametric models as recommended by statistical experts.

Missingness is not anticipated owing to the study design, but if it occurs at a meaningful level (>5%), we will perform sensitivity analysis using Multiple Imputation by Chained Equations to explore how missingness affects the results, and in the case that the results differ, we will report Multiple Imputation by Chained Equations as the primary analysis.

### Data and Code Availability

Raw data will be included as a digital supplement in each format needed to execute the included code (eg, .csv and .sav) alongside the full paper when published. All code needed to replicate the specific data cleaning and analysis steps in this paper will be included as a digital supplement alongside the full paper when published.

## Results

This protocol was prepared in full and submitted for review prior to any data collection or subject recruitment. The study will be conducted without using any external funding mechanisms. Results are expected to be published in late 2022 or early 2023.

## Discussion

### Expected Findings

This study is designed to determine whether the use of normative language in addition to cognitive language has an effect on a variety of measures related to general and specific trust in science and scientists as well as the credibility of the claims and their author(s). As indicated previously, we expect to see lower levels of general and localized trust in science and as well as reduced perceptions of credibility of both the post and the scientist(s) conducting the study among study participants who are exposed to both a cognitive and normative claim about a study than those exposed only to a cognitive claim.

### Next Steps

If 1 or more of our hypotheses are correct, then we hope to use these findings to initiate discussions about research dissemination and communication and the ways in which communication about research findings might more closely attend to and explicitly draw distinctions between cognitive and normative claims. This discussion may include conversations around changing the ways in which scientists communicate generally and addressing the complexity of separating normative and cognitive claims when communicating outside of the field (eg, with the media or policy makers).

We plan to disseminate the results of this study regardless of the findings through peer-reviewed publications as well as other avenues, as appropriate.

### Limitations

We anticipate that this study design may result in several limitations to its interpretation. First, we cannot control the conditions in which people participate; thus, although we will incorporate comprehension checks for the intervention, we cannot be assured that all participants will sufficiently engage with the intervention content. Second, since this is a web-based study through the Prolific platform, even though the results have a degree of national US generalizability, they still reflect the unique characteristics of those who have signed up for the Prolific platform as well as, by extension, those who actively use the internet. In a recent protocol for our COVID-19 intervention study, we outline why we believe these concerns are somewhat attenuated for this type of experiment [44]. Other limitations may be identified during the study itself or by reviewers at each stage of the project and will be noted after their identification.

## Data Availability

Data sharing is not applicable to this article as no data sets were generated or analyzed in the preparation of this protocol.
